# Beyond Self-Reports: Leveraging Multimodal Monitoring to Understand Caregiver Burden for Persons With Dementia

**DOI:** 10.1093/geroni/igaf122.754

**Published:** 2025-12-31

**Authors:** Kyung Hee Lee, Bomgyeol Kim, Jiyoung Shin

**Affiliations:** Yonsei University, Seodaemun-gu, Seoul-t’ukpyolsi, Republic of Korea; Yonsei University College of Nursing, Seodaemun-gu, Seoul-t’ukpyolsi, Republic of Korea; Health Insurance Research Institute, Wonju, Kangwon-do, Republic of Korea

## Abstract

Although numerous studies have examined the determinants of caregiver burden for people with dementia, the measurement of this burden has predominantly relied on self-report methods. The aim of this study was to identify the key determinants of the burden among family caregivers dealing with people with dementia using a multimodal monitoring approach. An observational cross-sectional study was conducted with 75 dyads of individuals with dementia and their caregivers. The determinants of caregiver burden were assessed within a biopsychosocial framework—encompassing biological, psychological, and social factors—using sweat patches, actigraphy, and surveys. A hierarchical multiple regression analysis was used to examine the associations. Psychological factors explained 39.3% of the variance in this burden, and an additional 17.5% increase was observed when biological factors were included, accounting for 56.8% of the variance. Higher levels of the pro-inflammatory cytokine TNF-α in persons with dementia were significantly associated with an increased caregiver burden, whereas longer total sleep time was linked to a decreased burden. Among the psychological factors, higher depressive symptoms and more severe behavioral and psychological symptoms of dementia were significantly related to an increased caregiver burden. Our findings underscore the need to integrate objective physiological measures with survey-based assessments to gain a more comprehensive understanding of caregiver burden. Furthermore, this study highlights the importance of tailored interventions that address both biological and psychological factors to alleviate caregiver burden and improve care outcomes. Reducing caregiver burden may also facilitate aging in place, enabling individuals with dementia to remain in familiar environments.

